# Cognitive Control and Ruminative Responses to Stress: Understanding the Different Facets of Cognitive Control

**DOI:** 10.3389/fpsyg.2021.660062

**Published:** 2021-05-07

**Authors:** Bita Zareian, Jessica Wilson, Joelle LeMoult

**Affiliations:** Depression, Anxiety and Stress Lab, Department of Psychology, The University of British Columbia, Vancouver, BC, Canada

**Keywords:** rumination, cognitive control, inhibition, shifting, updating, depression

## Abstract

Rumination has been linked to the onset and course of depression. Theoretical models and empirical evidence suggest that deficits controlling negative material in working memory underlie rumination. However, we do not know which component of cognitive control (inhibition, shifting, or updating) contributes most to rumination, and whether different components predict the more maladaptive (brooding) versus the more adaptive (reflection) forms of rumination. We aimed to advance theory and research by examining the contribution of different facets of cognitive control to the level and trajectory of brooding and reflection. At baseline, participants completed three cognitive tasks that assessed their inhibition, shifting, and updating biases, respectively. Next, using experience sampling methodology, participants rated their level of rumination and negative affect nine times during the 48 h after their most stressful exam. At each time point, higher levels of brooding, but not reflection, predicted higher levels of negative affect at the next time point. Furthermore, several facets of shifting and inhibition, but not updating, predicted brooding immediately after the exam and its trajectory of change over 48 h. Additionally, difficulty inhibiting neutral words predicted both brooding and reflection. These findings inform theoretical models describing the role of cognitive control in brooding and reflection.

## Introduction

Stressful life events, and our response to them, are one of the best predictors of depressive symptoms (Michl et al., 2013). Rumination is one way of responding to stress that has been shown to accelerate the transition from feeling distressed to feeling depressed (Bastin et al., 2015; Connolly and Alloy, 2017). Rumination is typified by repetitively and passively thinking about one’s problems, their causes, and their consequences (Nolen-Hoeksema, 1991). Evidence from both cross-sectional, longitudinal, and experience sampling (ESM) studies indicates that individuals who ruminate in response to stressful events endorse more depressive symptoms and are more likely to experience the onset of a depressive episode (Aldao et al., 2010; Ruscio et al., 2015).

However, not all types of rumination contribute equally to the development of depression. More recent conceptualizations of rumination distinguish between two different types of rumination: brooding and reflection (Treynor et al., 2003). Brooding is a more maladaptive form of rumination characterized by a passive and negative comparison of oneself with an unachieved ideal. Reflection, on the other hand, is a more adaptive form of rumination characterized by intentionally engaging in problem-solving to improve one’s depressive symptoms (Treynor et al., 2003). Researchers have documented that brooding and reflection have a different effect on depression (Treynor et al., 2003; Joormann et al., 2006; Burwell and Shirk, 2007). For instance, Treynor et al. (2003) found that participants who reported higher levels of brooding at baseline were more likely to feel depressed one year later. In contrast, those who reported higher levels of reflection at baseline were less likely to feel depressed one year later. Extending these findings, Moberly and Watkins (2008) used ESM to assess the moment-by-moment coupling between rumination and negative affect over time. They found that brooding, but not reflection, at each time point predicted negative affect at the future time point. Not only did these results document the differential effects of brooding versus reflection on negative affect, but the ESM also allowed researchers to establish a specific and immediate link between brooding and negative affect.

Despite the many studies that focus on the consequences of rumination and its subtypes, relatively few studies examine the mechanisms underlying ruminative responses to stress, and even fewer examine the different mechanisms underlying brooding versus reflection. Contemporary conceptualizations of rumination maintain that difficulty controlling negative material in working memory is a primary mechanism underlying ruminative responses to stress (Joormann and D’Avanzato, 2010; Joormann and Tanovic, 2015). Cognitive control functions play a central role in controlling the contents of working memory (Hasher and Zacks, 1988). Working memory has a restricted capacity and provides access to a limited number of mental representations at each point of time (Hasher and Zacks, 1988). Given the restricted capacity of working memory, cognitive control processes are tasked with regularly preventing irrelevant material from entering working memory and/or with updating the content of working memory as focus changes (Hasher et al., 1999).

Consistent with cognitive models of rumination (Joormann and Tanovic, 2015), researchers have documented that deficits in controlling negative material in working memory are associated with levels of trait rumination; specifically, individuals who struggle with inhibiting negative thoughts or with removing negative thoughts from working memory are more likely to engage in rumination following stress [as reviewed by Zetsche et al. (2018) and LeMoult and Gotlib (2019)]. However, there are several gaps in the extant literature. For one, previous research has often defined cognitive control as a unitary construct, when in fact, it is composed of three different processes: inhibition (i.e., suppressing task-irrelevant thoughts from entering working memory); shifting (i.e., switching between tasks and response rules); and updating (i.e., replacing previously relevant information in working memory with newer and more relevant information; Miyake and Friedman, 2012). There is evidence that biases in each of these subtypes of cognitive control are linked to rumination, and to brooding in particular. For instance, Joormann and Gotlib (2010) found that depressed participants who exhibited more difficulty inhibiting negative information from working memory reported higher levels of rumination, and they documented that negative inhibition biases were associated with self-reported levels of brooding, but not reflection. Rumination has also been associated with negative shifting biases, characterized by struggling *more* with switching away from the emotional meaning of *negative* stimuli, and struggling *less* with switching away from *positive* stimuli (Genet et al., 2013). While no studies to date have assessed the contribution of negative shifting biases to brooding versus reflection, previous studies provide evidence that difficulty in non-valenced shifting is associated with brooding, but not reflection (Whitmer and Banich, 2007; De Lissnyder et al., 2010). Finally, higher levels of rumination have been associated with difficulty updating negative information in working memory (Meiran et al., 2011). Although each facet of cognitive control has been linked to rumination, specifically to brooding, no study to date has simultaneously assessed the relative contribution of deficits in all three cognitive control components in relation to rumination, and more specifically to brooding versus reflection.

The second gap in the extant literature is that, to date, research that examines the association between cognitive control and rumination has focused on levels of trait rumination despite important evidence that levels of rumination fluctuate within a person over time (Moberly and Watkins, 2008). Theoretical models of rumination posit that rumination increases during times of stress (Nolen-Hoeksema et al., 2008). Hence, while previous studies provide us with evidence on the association between deficits in cognitive control and trait rumination, the role of these cognitive control deficits on the moment-to-moment fluctuations in rumination has rarely been investigated (Hoorelbeke et al., 2016). Given that ESM is ideally positioned to capture these moment-to-moment changes in rumination as well to assess the coupling between levels of rumination and negative affect, we took advantage of ESM to address our research questions.

The current study was designed to address these gaps in the literature by assessing the relative association of the three components of cognitive control with brooding and reflection in response to a stressful event. Given the pernicious role of rumination in the exacerbation of depression in unselected samples (Connolly and Alloy, 2017), and given the fact that university students are particularly vulnerable to depression (Ibrahim et al., 2013), we recruited an unselected sample of undergraduate students. Participants came into the lab for a baseline session, during which they completed three cognitive tasks, each of which assessed one component of cognitive control: inhibition, shifting, and updating. Next, we used ESM to assess participants’ level of brooding, reflection, and affect for 48 h following a commonly occurring naturalistic stressor shown previously to elicit rumination: a midterm exam (Grant and Beck, 2010).

We first aimed to replicate the finding that brooding, and not reflection, at each time point predicts the level of negative affect at the next time point (Aim 1; Moberly and Watkins, 2008). Next, we aimed to assess the relative association of each component of cognitive control on the initial level and subsequent trajectory of brooding and reflection (Aim 2). Because this is the first study to assess the relative contribution of all three components of cognitive control on rumination, we made no *a priori* hypothesis about which component would best predict the level and trajectory of rumination. However, based on theoretical models and empirical evidence demonstrating that cognitive control is associated with brooding, but not reflection (Whitmer and Banich, 2007; De Lissnyder et al., 2010), we expected that cognitive control would predict the level and trajectory of brooding, and not reflection.

## Materials and Methods

### Participants

We recruited a sample of 268 participants through the University of British Columbia Human Subject Research Pool. Participants were excluded if they were color blind. Based on recommendation of Maas and Hox (2005), we recruited a sample larger than 50 to have adequate power to analyze the data using a two level Hierarchical Linear Model (HLM). We did not constrain the maximum sample size; rather, we included all participants in our sample who participated during the course of two semesters; we halted recruitment at the end of the second term before any data were analyzed. Data from 19 participants could not be included in the final analyses because of errors in determining the time of their exam. In addition, 20 participants did not complete any of the nine follow-up surveys. Thus, 229 participants were included in the final sample. Level of depression at baseline did not differ between those who were and were not in the final analyses, *t*(265) = −1.35, *p* = 0.178. The average age of participants was 20.34 years (*SD* = 2.61) and the majority were female (82%). Participants were from diverse ethnic backgrounds: 59% identified as East Asian, 25% as Caucasian, and 16% as belonging to other ethnicities. Demographic information is summarized in Table 1.

**TABLE 1:** Demographic characteristics.

Variables	%
**Sex**	
Male	16
Female	83
Other	1
**Ethnicity**	
Asian	59
Caucasian	25
Other	16
**Year in school**	
1st	24
2nd	24
3rd	31
4th	15
≥5th	6

### Measures

#### Rumination

We used the short form of the Ruminative Response Scale (RRS; Treynor et al., 2003) to assess participants’ self-reported level of rumination at each follow-up assessment. The questions themselves were kept in their original format. However, we modified the questionnaire instructions to assess the level of state rumination since their last survey or midterm exam (depending on the follow-up). Specifically, in the first follow-up, we asked participants to assess their level of rumination since the exam (i.e., please read each of the following items and think about the time between now and your midterm exam). In the remaining eight follow-ups (i.e., the 2nd to 9th follow-up survey), we asked participants to assess their level of rumination since they last responded to one of the study surveys (i.e., please read each of the following items and think about the time between now and when you last completed the survey). Both brooding and reflection subscales have demonstrated good test-retest reliability, and brooding has shown particularly good predictive validity for current and future depressive symptoms (Treynor et al., 2003). In the current study, αs = 0.84–0.92; ICC = 0.68.

#### Negative Affect

We used negative affect items taken from the Brief Positive and Negative Affect Schedule (PANAS; Watson et al., 1988; Watson and Clark, 1999) to assess the intensity of participants’ negative affect at each follow-up assessment. The negative affect score was calculated by averaging participants’ ratings of guilt, anxiety, anger, shame, sadness, upset, and tension (αs = 0.91–0.94; ICC = 0.57).

#### Depression

Participants completed the Center for Epidemiologic Studies Depression Scale (CES-D; Radloff, 1977), which assesses the frequency of depressive symptoms experienced in the past week. We asked participants to complete the CES-D at the baseline session in order to control for the effect of baseline depression (α = 0.77).

### Cognitive Tasks

We used three different cognitive tasks, Emotional Stroop Task (Gotlib and McCann, 1984), Affective Switching Task (Genet et al., 2013), and Emotional 2-Back Task (Levens and Gotlib, 2012), to assess inhibition, shifting, and updating, respectively. All three tasks were programmed in E-prime 2, and are described below.

#### Inhibition

Based on factor-analytic results (Friedman et al., 2008) and evidence of strong psychometric properties (Eide et al., 2002), we chose the Emotional Stroop Task (Gotlib and McCann, 1984) to assess inhibition biases. We included six different trial types: positive, negative, threatening, neutral, same-color (i.e., color word presented in the same color font), and different-color (i.e., color word presented in a different color font). Each trial began with a fixation cross presented for 500 ms on a black screen. Next, participants viewed words one at a time. Each word was presented in a different color (i.e., blue, yellow, and green), and participants indicated the color of the word. The words were chosen from those validated by Gotlib et al. (2004). In total, participants completed 135 experimental trials (27 of each word category). Consistent with past research (Sharma and McKenna, 2001; Mitterschiffthaler et al., 2008), experimental trials were presented for all participants in the same pseudorandom sequence, constrained by not presenting more than two consecutive words from the same category or color. The word itself was determined at random for each participant.

Consistent with past research (Eide et al., 2002), we calculated participants’ average reaction time on accurate trials for each trial type. The task showed adequate psychometric properties, with split-half Spearman-Brown corrections ranging from 0.87 to 0.92 for each trial type. Same-color and different-color trials used in the original (non-Emotional) Stroop Task were used in the current study to replicate the well-established finding that participants show greater interference on different-color trials than same-color trials (Stroop, 1992; see Supplementary Material). The other four trial types (i.e., positive, negative, threat, and neutral) are part of the Emotional Stroop Task (Gotlib and McCann, 1984), and were used to assess inhibition. The more difficulty participants have inhibiting the meaning of a word, the longer it would take for them to indicate the color in which the word is written. Thus, greater reaction times on positive and negative trials indicate positive and negative inhibition biases, respectively.

#### Shifting

Given our interest in assessing shifting biases toward positive and negative affective material, we chose the Affective Switching Task (Genet et al., 2013). In this task, participants viewed pictures that have either a negative or a positive valence, and then sorted each picture based on either an affective rule [i.e., positive (+) versus negative (−)] or a non-affective rule [i.e., one or less human in the picture (≤1) versus two or more humans in the picture (≥2)].

Each trial started with a fixation cross presented for 250 ms, followed by a blank screen for another 250 ms. Participants then saw an emotional picture and the sorting rule for that trial, both of which remained on the screen until the participant’s response was logged. Pictures were chosen from the International Affective Picture Set (IAPS; Lang, 2005), and we used the same 180 images used by Genet et al. (2013). The combination of picture valence (i.e., + or −) and the number of humans in the picture (≤1 or ≥2) creates a total of four picture categories. Participants first completed two 10-trial practice blocks to become familiar with the task, and then completed two 160-trial experimental blocks (40 trials from each picture category). We used the same pseudorandom trial order and images (presented at random within each category) used by Genet et al. (2013).

Participants’ reaction time and accuracy were logged for each of the four picture categories (see Supplementary Material for additional details). As described in Genet et al. (2013), from these data, four switch costs were calculated to assess switching biases: affective positive switch, non-affective positive switch, affective negative switch, non-affective negative switch. The task showed adequate psychometric properties, with split-half Spearman-Brown corrections ranging from 0.82 to 0.93 for each type of switch cost trial. Greater affective switch costs reflect more difficulty switching focus away from non-valenced aspects of information. Greater non-affective switch costs reflect more difficulty switching focus away from valenced aspects of information. As a result, the two non-affective switch costs, non-affective positive switch and non-affective negative switch, are indicative of positive and negative shifting biases, respectively.

#### Updating

Based on factor-analytic results (Friedman et al., 2008) and evidence of strong psychometric properties (Soveri et al., 2018), we used an affective version of the 2-Back Task (Levens and Gotlib, 2012) to assess updating biases. Each trial began with a fixation cross presented for 2,250 ms on a blank screen. Next, participants viewed images of a facial expression of emotion (happy, neutral, sad) one at a time, and indicated whether the valence of the face was the same as or different from the face seen two images earlier. Stimuli used in the Affective 2-Back Task came from the MacArthur Network Face Stimuli Set1^1^, which was developed by The Research Network on Early Experience and Brain Development. We used the same 138 images used by Levens and Gotlib (2012): 46 sad, 46 neutral, and 46 happy faces. In total, participants completed 278 trials divided into five separate experimental blocks, and faces were presented in the same pseudorandom order within each experimental block. The task showed adequate psychometric properties, with split-half Spearman-Brown corrections ranging from 0.73 to 0.88 for each trial type.

Given that Levens and Gotlib (2010, 2015) preferred the use of reaction time for this task and that Dai et al. (2015) found that reaction time (and not accuracy), was associated with rumination levels, we used reaction time on different types of trials as the outcome variable for this task. There are three different trial types in this task: match-set, break-set, and no-set. In match-set trials, participants see the same facial expression that they had seen two faces before. Break-set trials are trials that are presented right after a match-set trial and show a different facial expression than the two trials before. In break-set trials, participants have to break the previous set to respond to the current trial. Finally, in no-set trials, participants see a facial expression that does not match two faces before, and participants are not required to break a set. Among these different trial types, break-set trials are of particular importance in assessing updating. Longer reaction times on break-set trials indicate that the participant is struggling with expelling the previous set and requires more time to update the content of their working memory to respond to the current trial. In other words, break-set trials (i.e., Break-sad, Break-happy, Break-neutral) for each valence (happy, neutral, sad) assess participants’ ability to update no longer relevant information with newer and more relevant information. Thus, reaction times on accurate break-set trials are considered to be the best metric of updating biases (Levens and Gotlib, 2015).

### Procedure

We obtained approval for this study from the Behavioral Research Ethics Board. In the first lab session, participants provided informed consent and then completed the three cognitive tasks. The order in which the tasks were completed was counterbalanced across participants. Consistent with prior research (Scher et al., 2005; LeMoult et al., 2017), participants watched a negative movie clip to induce a negative mood state before each cognitive task. Inducing negative affect prior to completing cognitive tasks is used in the lab to parallel the activation of cognitive biases that occurs in response to negative mood states in everyday life. This is in keeping with theoretical models of cognition and depression (Teasdale, 1988) and with empirical evidence (Schoofs et al., 2008; Quinn and Joormann, 2015) that suggest that cognitive biases remain dormant, unless triggered by negative mood states. To ensure that participants’ negative affect increased following the movie clips, participants reported on their negative affect before and after each movie clip (see Supplementary Material). After completing the three cognitive tasks, participants completed questionnaires about their demographics, and frequency of depressive symptoms. They were also asked to provide the time and date of their most stressful midterm exam. Given that this study was part of a larger project, participants also completed questionnaires that assessed anxiety, trauma, social support, resilience, and other facets of emotion regulation. These measures were not analyzed given that they were not relevant to the *a priori* hypotheses reported here.

The average time between the baseline session and the time of participants’ most stressful midterm was approximately 19 days (*M* = 18.45, *SD* = 13.01). We used SurveySignal (Hofmann and Patel, 2015) to send nine follow-up surveys to participants at different time-points during the 48 h after this exam. The first survey was sent right after the exam, and the next eight surveys were sent using stratified random sampling with a minimum of 45 min between two consecutive surveys, which is consistent with best-practice recommendations (Verhagen et al., 2016). Surveys were sent between 9 AM and 9 PM so as not to disturb sleep patterns (see the Supplementary Material for more information). For all follow-up surveys, except for the last follow-up survey, the survey link was sent through text message, and it expired 2 h after it was sent to participants. The last follow up survey was sent over email, 48 h after the exam, and participants were asked to complete it as soon as possible. To receive course credit for the study, participants were required to complete at least five of the nine follow-up surveys, one of which had to be the last survey. In these nine follow-up surveys (i.e., follow-up 1 to 9), participants answered questions about their current level of rumination (i.e., state rumination) and negative affect.

### Statistical Analysis

We used HLM-7 (Raudenbush et al., 2011) to analyze the data for Aim 1 and Aim 2 using Hierarchal Linear Modeling (HLM). HLM offers several distinct advantages over other analytic methods (Raudenbush and Bryk, 2002), including allowing for unevenly spaced repeated measurements and accounting for missing data. These advantages are particularly useful in this study given that the interval between surveys was different for each participant and that participants were not required to respond to all of the surveys. Additionally, HLM allows all predictor variables to be entered within the same model, enabling the relative contribution of each predictor to be assessed simultaneously, thereby minimizing Type I error by eliminating the need for running multiple analysis. Prior to analyses, we standardized (i.e., z-scored) and grand-mean centered all Level 2 predictors. We further used the r2beta function from the r2glmm package (Jaeger, 2017) to calculate *R*^2^s for Level 2 predictors, in R 4.0.4 (R Core Team, 2021). As discussed in Snijders and Bosker (2011) and in previous studies (Carels et al., 2007), however, multilevel modeling is not favorable to traditional computations of effect size or proportion of variance explained (e.g., *R*^2^). As a result, the reported *R*^2^s should be interpreted with caution. The raw data and cognitive tasks are available upon request; full HLM equations and R-code are available in the Supplementary Material.

#### Aim 1: Brooding, and Not Reflection, Predicts Negative Affect at the Following Time Point

To examine whether brooding and reflection at each time point (*t*) predicted negative affect at the next time point (*t* + 1), we conducted a time-lagged hierarchal linear model. We examined whether brooding and reflection at time *t* predicted negative affect at time *t* + 1, controlling for negative affect at time *t*:

Level⁢ 1:Negative⁢Affect(t+1)⁢ij=β0⁢j+β1⁢j⁢(Broodingt)+β2⁢j⁢(Reflectiont)+β3⁢j⁢(Negative⁢Affectt)+etj

#### Aim 2: Deficits in Cognitive Control Predict the Level and Trajectory of Brooding and Reflection

We also used HLM to analyze the influence of baseline cognitive control deficits on participants’ level and trajectory of brooding and reflection after their exam. Analyses were run using Full Maximum Likelihood to assess model fit and Restricted Maximum Likelihood to test our hypotheses. We first tested linear, quadratic, and piecewise models (with no Level 2 predictors) to determine the model that best captured changes in brooding and reflection in response to a stressful exam (see Supplementary Material). We then entered at Level 2 the grand-mean centered standardized cognitive bias scores and baseline CES-D scores (as a covariate).

##### Brooding

**FIGURE 1:**
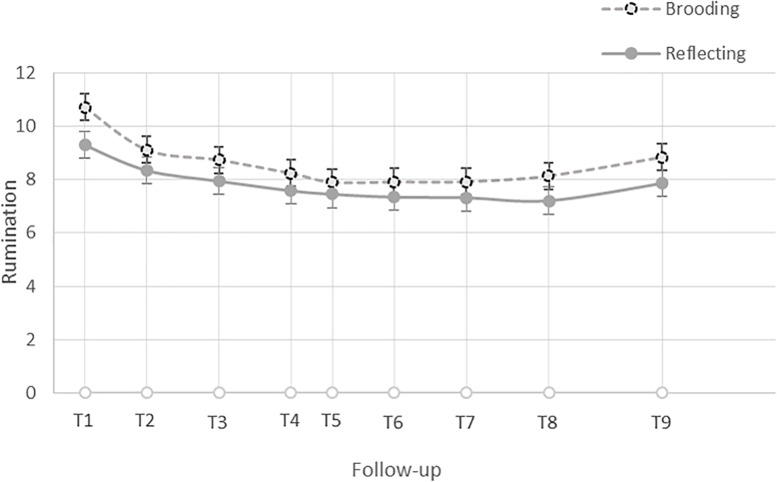
Trajectory of change in the level of brooding and reflection in the 48 h after participants’ exam.

In keeping with Raudenbush and Bryk (2002), we first visually inspected the data and found a discontinuous trajectory of change in levels of brooding in the 48 h following participants’ exam, which is consistent with a piecewise model. Preliminary analyses of the data, the Akaikie’s Information Criteria (AIC), and the deviance statistics, confirmed that the piecewise linear growth model was the best fit for the data. Specifically, participants’ average level of brooding was highest immediately after the exam, *B* = 9.99, *SE* = 0.267, *t*(187) = 37.36, *p* < 0.001, declined with a steep slope until an average of 8 h later (i.e., follow-up 2), *B* = −0.19, *SE* = 0.039, *t*(187) = −4.752, *p* < 0.001, and then subsequently declined with a flatter slope until the last survey, 48 h later (i.e., follow-up 9), *B* = −0.03, *SE* = 0.005, *t*(187) = −5.93, *p* < 0.001 (see Figure 1). Thus, at Level 1, we estimated brooding right after the exam (intercept), its initial trajectory of change until follow-up 2, and its trajectory of change after follow-up 2. We then entered the grand-mean centered standardized cognitive bias scores and baseline CES-D scores (as a covariate) as predictors at Level 2. See Supplementary Material for additional details.

##### Reflection

In order to test whether the associations between different facets of cognitive control and rumination were specific to brooding, we repeated the above steps using reflection as the outcome variable. Similar to brooding, visual inspection of the reflection data revealed a discontinuous trajectory of change in levels of reflection in the 48 h following participants’ exam, which is consistent with a piecewise model. Preliminary analyses of the data, the AIC, and the deviance statistics (see the Supplementary Material), confirmed that the piecewise linear growth model was the best fit for the data. Specifically, participants’ average level of reflection was significantly greater than zero after the exam, *B* = 8.70, *SE* = 0.229, *t*(187) = 38.04, *p* < 0.001, declined after the exam until an average 8 h later (i.e., follow-up 2) with a steep slope, *B* = −0.09, *SE* = 0.034, *t*(187) = −2.73, *p* = 0.007, and then declined with a flatter slope thereafter, *B* = −0.02, *SE* = 0.004, *t*(187) = −6.16, *p* < 0.001 (see Figure 1). Thus, at Level 1, we estimated reflection right after the exam (intercept), its initial trajectory of change until follow-up 2, and its trajectory of change after follow-up 2. We then entered the grand-mean centered standardized cognitive bias scores and baseline CES-D scores (as a covariate) as Level 2 predictors.

## Results

### Preliminary Analyses

Of the 229 participants in the final sample, participants completed an average of 6.79 surveys (*SD* = 1.93). The number of completed surveys was not associated with demographic characteristics. However, participants with higher levels of depression and rumination at baseline responded to fewer follow-up surveys, *r* = −0.15, *p* = 0.024, and *r* = −0.14, *p* = 0.038, respectively. After controlling for baseline depression levels, however, baseline rumination was no longer significantly associated with the number of follow-up surveys completed, *partial r* = −0.07, *p* = 0.297.

Participants’ demographic characteristics are presented in Table 1, and the first-order correlations among all cognitive variables are presented in Table 2 (for more information about cognitive tasks data cleaning see the Supplementary Material). Participants’ average score on the CES-D at baseline was 19.95 (*SD* = 7.57), which is above the recommended cut-off of 16 that indicates a clinically significant level of depressive symptoms (Radloff, 1977).

**TABLE 2:** Correlation table for cognitive variables.

	Cognitive variable	*M* (*SD*)	Correlation										
			
			1	2	3	4	5	6	7	8	9	10	11
1	Affective positive switch cost	77.25 (192.35)	–										
2	Affective negative switch cost	212.19 (184.41)	0.170**	–									
3	Non-affective positive switch cost	114.29 (179.17)	0.132*	0.098	–								
4	Non-affective negative switch cost	0.50 (217.00)	0.147*	0.138*	0.066	–							
5	Break-happy	1109.90 (118.01)	0.123	0.093	0.049	0.029	–						
6	Break-neutral	1106.29 (118.88)	0.083	0.065	0.115	0.106	0.745**	–					
7	Break-sad	1119.62 (116.00)	0.093	0.026	0.078	0.081	0.710**	0.769**	–				
8	Stroop-negative	627.29 (87.49)	0.144*	0.138*	0.050	0.049	0.272**	0.199**	0.211**	–			
9	Stroop-neutral	628.38 (82.45)	0.140*	0.149*	0.067	−0.035	0.252**	0.173**	0.181**	0.918**	–		
10	Stroop-positive	630.29 (85.67)	0.113	0.150*	0.023	0.016	0.288**	0.213**	0.232**	0.909**	0.902**	–	
11	Stroop-threat	640.52 (89.81)	0.139*	0.155*	0.042	0.029	0.266**	0.179**	0.209**	0.915**	0.894**	0.914**	–

*p < 0.05, **p < 0.01.

### Main Analyses

#### Aim 1: Brooding, and Not Reflection, Predicts Negative Affect at the Following Time Points

Consistent with our hypotheses, brooding at each time point, *B* = 0.19, *SE* = 0.078, *t*(212) = 2.38, *p* = 0.018, *R*^2^ = 0.01 predicted negative affect at the next time point (*t* + 1). In contrast, reflection at each time point (*t*) did not significantly predict negative affect at the next time point (*t* + 1), *B* = 0.072, *SE* = 0.091, *t*(212) = 0.79 *p* = 0.429, *R*^2^ = 0.002. Negative affect at each time point (*t*) also predicted negative affect at the next time point (*t* + 1) in the model, *B* = 0.341, *SE* = 0.036, *t*(212) = 9.45, *p* < 0.001, *R*^2^ = 0.17.

#### Aim 2: Deficits in Cognitive Control Biases at Baseline Predict the Level of Brooding and Reflection After the Exam and Their Trajectory of Change in the Next 48 h

##### Brooding

We ran a single HLM model to examine whether the level and trajectory of brooding were predicted by inhibition, shifting, and updating biases at baseline, after controlling for the baseline level of depressive symptoms. Results are presented in Table 3. As expected, higher levels of brooding immediately after the exam were predicted by higher levels of depression at baseline, *B* = 0.786, *t*(175) = 2.28, *p* = 0.024, *R*^2^ = 0.02. Furthermore, higher levels of brooding immediately after the exam were predicted by less positive shifting bias, more negative shifting bias, less positive inhibition bias, and less inhibition of neutral stimuli at baseline. Specifically, higher levels of brooding were associated with *faster* switching away from positive (i.e., less positive shifting bias), *B* = −0.662, *t*(175) = −2.26, *p* = 0.025, *R*^2^ = 0.02, and *slower* switching away from negative (i.e., more negative shifting bias), *B* = 0.813, *t*(175) = 3.00, *p* = 0.003, *R*^2^ = 0.02. In addition, higher levels of brooding immediately after the exam were associated with greater inhibition of positive (i.e., preventing positive words from entering working memory or positive inhibition bias), *B* = −1.930, *t*(175) = −2.54, *p* = 0.012, *R*^2^ = 0.02, and less inhibition of neutral (i.e., preventing neutral words from entering working memory), *B* = 2.058, *t*(175) = 2.70, *p* = 0.008, *R*^2^ = 0.02.

**TABLE 3:** Predicting the level and trajectory of brooding.

	Coeff	SE	*t* (175)	*p*
**Intercept**				
Intercept	**10.75**	**0.301**	**35.72**	**<0.001**
Baseline depression	**0.79**	**0.345**	**2.28**	**0.024**
Stroop-negative	−1.287	0.810	−1.59	0.114
Stroop-neutral	**2.058**	**0.763**	**2.70**	**0.008**
Stroop-positive	−**1.930**	**0.760**	−**2.54**	**0.012**
Stroop-threat	1.563	0.960	1.63	0.105
Affective positive switch cost	−0.030	0.326	−0.09	0.927
Affective negative switch cost	0.257	0.283	0.91	0.365
Non-affective positive switch cost	−**0.662**	**0.293**	−**2.26**	**0.025**
Non-affective negative switch cost	**0.813**	**0.271**	**3.00**	**0.003**
Break-happy	0.234	0.512	0.46	0.648
Break-neutral	−0.281	0.540	−0.52	0.603
Break-sad	0.187	0.485	0.39	0.700
**Slope of change until follow-up 2**				
Intercept	−**0.455**	**0.077**	−**5.94**	**<0.001**
Baseline depression	0.059	0.075	0.79	0.433
Stroop-negative	0.313	0.218	1.44	0.153
Stroop-neutral	−**0.522**	**0.179**	−**2.92**	**0.004**
Stroop-positive	**0.477**	**0.181**	**2.63**	**0.009**
Stroop-threat	−0.348	0.255	−1.36	0.175
Affective positive switch cost	0.045	0.069	0.65	0.517
Affective negative switch cost	0.005	0.055	0.10	0.924
Non-affective positive switch cost	**0.236**	**0.084**	**2.82**	**0.005**
Non-affective negative switch cost	−**0.172**	**0.062**	−**2.79**	**0.006**
Break-happy	−0.136	0.103	−1.33	0.187
Break-neutral	0.236	0.133	1.78	0.078
Break-sad	−0.139	0.106	−1.31	0.193
**Slope of change after follow-up 2**				
Intercept	−**0.027**	**0.005**	−**5.06**	**<0.001**
Baseline depression	0.001	0.008	0.15	0.878
Stroop-negative	0.022	0.017	1.34	0.182
Stroop-neutral	−0.025	0.013	−1.96	0.052
Stroop-positive	0.016	0.016	1.00	0.320
Stroop-threat	−0.013	0.020	−0.65	0.516
Affective positive switch cost	0.003	0.004	0.58	0.565
Affective negative switch cost	−0.001	0.005	−0.27	0.789
Non-affective positive switch cost	−0.001	0.004	−0.349	0.727
Non-affective negative switch cost	−**0.011**	**0.005**	−**2.351**	**0.020**
Break-happy	−0.0001	0.010	−0.02	0.988
Break-neutral	−0.005	0.008	−0.59	0.557
Break-sad	0.003	0.008	0.34	0.732

*The bolded values indicate that the values are statistically significant.*

The slope of decline in brooding from immediately after the exam until the second follow-up, which was on average 8 h after the exam, was predicted by the same baseline cognitive variables that predicted the initial level of brooding, but in the opposite direction: more sustained brooding was associated with more positive shifting bias, less negative shifting bias, more positive inhibition bias, and more inhibition of neutral stimuli. Specifically, a flatter slope of decline in brooding was associated with slower switching away from positive (i.e., positive shifting bias), *B* = 0.236, *t*(175) = 2.82, *p* = 0.005, *R*^2^ = 0.09, and more difficulty inhibiting positive (i.e., positive inhibition bias), *B* = 0.477, *t*(175) = 2.63, *p* = 0.009, *R*^2^ = 0.06. A flatter slope of decline in brooding was also associated with faster switching away from negative (i.e., negative shifting bias), *B* = −0.172, *t*(175) = −2.79, *p* = 0.006, *R*^2^ = 0.07, and more inhibition of neutral, *B* = −0.522, *t*(175) = −2.923, *p* = 0.004, *R*^2^ = 0.08. Finally, the slope of change after the second follow-up continued to be predicted by negative shifting bias: more sustained brooding after the second follow-up was associated with faster switching away from negative, *B* = −0.011, *t*(175) = −2.35, *p* = 0.020, *R*^2^ = 0.004. Adding the time between the baseline session and the exam as a covariate did not change the results.

##### Reflection

We ran a second HLM model using reflection as the outcome variable to examine whether reflection right after the exam and its slope of change were predicted by inhibition, shifting, and updating biases at baseline, controlling for the baseline level of depression (see Table 4). Reflection right after the exam was predicted by only difficulty inhibiting neutral stimuli, *B* = 0.166, *t*(175) = 2.55, *p* = 0.012, *R*^2^ = 0.02, such that greater reflection immediately after the exam was associated with less inhibition of neutral. The slope of change in reflection from immediately after the exam until the second follow-up, which was on average 8 h after the exam, was associated with positive shifting bias, *B* = 0.148, *t*(175) = 2.16, *p* = 0.032, *R*^2^ = 0.06, and inhibition of neutral stimuli, *B* = −0.596, *t*(175) = −3.98, *p* < 0.001, *R*^2^ = 0.15, such that a flatter slope of decline in reflection was associated with slower switching away from positive and less difficulty inhibiting neutral. The slope of change after the second follow-up was not predicted by any of the cognitive variables. Adding the time between the baseline session and the exam as a covariate did not change the results.

**TABLE 4:** Predicting the level and trajectory of reflection.

	Coeff	SE	*t* (175)	*p*
**Intercept**				
Intercept	**9.266**	**0.253**	**36.56**	**<0.001**
Baseline depression	0.401	0.282	1.42	0.157
Stroop-negative	−0.876	0.656	−1.34	0.184
Stroop-neutral	**1.660**	**0.650**	**2.55**	**0.012**
Stroop-positive	−1.091	0.677	−1.61	0.109
Stroop-threat	0.915	0.882	1.04	0.301
Affective positive switch cost	−0.215	0.282	−0.76	0.446
Affective negative switch cost	0.404	0.237	1.70	0.090
Non-affective positive switch cost	−0.134	0.247	−0.54	0.589
Non-affective negative switch cost	0.404	0.244	1.66	0.099
Break-happy	0.314	0.391	0.80	0.423
Break-neutral	−0.520	0.416	−1.25	0.213
Break-sad	0.477	0.365	1.31	0.193
**Slope of change until follow-up 2**				
Intercept	−**0.292**	**0.064**	−**4.552**	** < 0.001**
Baseline depression	0.065	0.068	0.96	0.337
Stroop-negative	0.389	0.202	1.92	0.056
Stroop-neutral	−**0.596**	**0.150**	−**3.98**	**<0.001**
Stroop-positive	0.352	0.180	1.95	0.052
Stroop-threat	−0.310	0.237	−1.31	0.193
Affective positive switch cost	0.060	0.057	1.07	0.287
Affective negative switch cost	−0.004	0.053	−0.07	0.943
Non-affective positive switch cost	**0.148**	**0.068**	**2.16**	**0.032**
Non-affective negative switch cost	−0.100	0.058	−1.74	0.085
Break-happy	−0.042	0.085	−0.49	0.626
Break-neutral	0.174	0.103	1.69	0.094
Break-sad	−0.171	0.091	−1.88	0.062
**Slope of change after follow-up 2**				
Intercept	−**0.022**	**0.004**	−**5.723**	**<0.001**
Baseline depression	−0.006	0.005	−1.18	0.240
Stroop-negative	0.013	0.013	0.99	0.322
Stroop-neutral	−0.009	0.010	−0.90	0.367
Stroop-positive	0.017	0.014	1.27	0.206
Stroop-threat	−0.015	0.013	−1.18	0.240
Affective positive switch cost	0.001	0.004	0.15	0.880
Affective negative switch cost	0.00003	0.004	0.01	0.994
Non-affective positive switch cost	−0.006	0.003	−1.88	0.062
Non-affective negative switch cost	−0.006	0.004	−1.57	0.118
Break-happy	−0.002	0.008	−0.33	0.745
Break-neutral	−0.001	0.006	−0.23	0.822
Break-sad	−0.0002	0.006	−0.03	0.976

*The bolded values indicate that the values are statistically significant.*

## Discussion

To our knowledge, this is the first study to assess the relative contribution of the three components of cognitive control– inhibition, shifting, and updating – to rumination. We used an ESM design to model the level and trajectory of rumination after a naturalistic stressor. We then examined the relative contribution of deficits in inhibition, shifting, and updating to brooding and reflection. We found that brooding at each time point predicted negative affect at the next time point. We also found that several facets of shifting and inhibition, but not updating, predicted brooding immediately after the exam and its trajectory of change over the course of 48 h.

Replicating previous research that has used ESM (Moberly and Watkins, 2008), we found that brooding, and not reflection, predicted higher levels of negative affect at the next sampling occasion. These results are also consistent with findings of previous longitudinal studies showing that brooding predicts the onset and worsening of depression six to 12 months later (Treynor et al., 2003; Burwell and Shirk, 2007). Some longitudinal studies have shown a protective effect of reflection at baseline on the development of depressive symptoms (Treynor et al., 2003; Burwell and Shirk, 2007). These studies have used a follow-up of six months or more, which stands in contrast to the shorter time course examined in the current study. It is possible that while the consequences of brooding on depressive symptoms are detectable early on as a result of its direct effect on negative affect, the protective effect of reflection becomes evident later on, resulting from an increased capacity to problem solve over time.

We also assessed the relative contribution of deficits in inhibition, shifting, and updating information on the level and trajectory of brooding and reflection following a stressor. Previous theoretical models of rumination have conceptualized cognitive control as a unitary construct. We, however, introduced a more nuanced perspective on the association between cognitive control and rumination by assessing the relative contribution of biases within the three cognitive control components: inhibition, shifting, and updating (Miyake and Friedman, 2012). Interestingly, we found that several facets of shifting and inhibition, but not updating, predicted brooding immediately after the exam and its trajectory of change over the course of 48 h. More specifically, we found that participants who had more difficulty inhibiting neutral information and less difficulty inhibiting positive information reported higher levels of brooding immediately after the exam. In addition, we found that more negative shifting biases (i.e., more difficulty shifting away from the valenced aspect of negative stimuli) and less positive shifting biases (i.e., less difficulty shifting away from the valenced aspect of positive stimuli) were associated with higher levels of brooding immediately after the exam. In other words, the “more sticky” the negative information and the “less sticky” the positive information is for an individual, the more likely individuals were to brood after a stressful event. In contrast, updating biases were not significantly associated with the level or trajectory of brooding, which is contrary to empirical evidence showing that negative updating biases have a stronger influence on levels of rumination (Meiran et al., 2011; Pe et al., 2013). This discrepancy could be the result of using affective stimuli rather than the more frequently used neutral stimuli. The majority of previous studies have used neutral stimuli when assessing the association between rumination and cognitive control, in general, and rumination and updating in particular (Friedman et al., 2008). Therefore, while there might be an association between “cold cognition” updating and rumination, this association might not hold when using affective stimuli. In other words, while individuals who ruminate might find it difficult to update information in general, they might not demonstrate positive or negative updating biases. That being said, previous studies have found an association between updating biases and rumination (Meiran et al., 2011). Therefore, it is also possible that the association between updating and rumination found in previous research may have been influenced by the overlap between updating biases with other components of cognitive control. This formulation is supported by neuroimaging studies that show the same brain regions are responsible for multiple aspects of cognitive control. For example, although there are specific areas of the prefrontal cortex (PFC) linked to each facet of cognitive control (Levy and Wagner, 2011), there are other areas of the PFC (e.g., the inferior frontal junction) recruited by all three cognitive control components (Derrfuss et al., 2004). In order to see if this possibility was supported by our data, we conducted exploratory analyses in which we entered the n-back task variables as the sole predictor of the level and trajectory of rumination after the exam. We found that, in fact, without controlling for other components of cognitive control, more difficulty updating neutral material in WM is associated with a steeper slope of change in rumination right after the exam (see the Supplementary Material).

Interestingly, difficulty inhibiting neutral words was associated with both brooding and reflection immediately after the exam. It is important to consider why difficulty processing neutral words, in contrast to positive or negative words, might be associated with both brooding and reflection. One possible reason is that difficulty inhibiting neutral information reflects a form of “cold” cognition (Roiser and Sahakian, 2013) that underlies both adaptive and maladaptive types of repetitive cognition. This possibility is supported by results from previous studies that document difficulty with inhibition is associated with both brooding and reflection (Whitmer and Banich, 2007), and that this difficulty is not necessarily related to the valence of information presented in the inhibition task (Goeleven et al., 2006). These results are also consistent with findings that deficits in cold cognition are linked to other repetitive patterns of thought such as worry and obsessions (for review see Hallion et al., 2019).

We also found that the trajectory of change in brooding was predicted by the same variables that predicted the level of brooding right after the exam, but in the opposite direction: faster recovery of brooding from the time of the exam to the second follow-up was predicted by more difficulty inhibiting neutral information, less difficulty inhibiting positive information, more negative and less positive shifting biases. We found a similar direction of effects for the cognitive control deficits that predicted the trajectory of change in reflection. Interestingly, in our data, coefficients estimating levels of brooding at the first time point just after the exam (i.e., the intercept) were negatively correlated with coefficients estimating the change in brooding following the exam. Thus, one possible explanation for the unexpected direction of findings predicting the trajectory of change in brooding is that our stressor, a stressful midterm exam, did not induce adequate stress that would result in sustained levels of rumination over a 2 day period. As such, our results might reflect a regression to the mean, in which regardless of level of rumination after the exam, all participants went back to a baseline level of rumination within 8 h after the exam. In other words, those with higher levels of rumination right after the exam also had a steeper slope of decline after the exam. As a result, higher negative cognitive biases (and lower positive cognitive biases) are predictors of both higher levels of brooding and reflection right after the exam, and a steeper slope of decline in these emotion regulation strategies over the course of 8 h after the exam. Another possible explanation for these results is that participants with more difficulty controlling negative material (or less difficulty controlling positive material) are more reactive to stress, evidenced by higher levels of rumination immediately after the exam followed by a steeper decline back to baseline. This proposition is supported by findings suggesting that those who demonstrate cognitive biases are more emotionally reactive to stress (Fox et al., 2010) and have difficulty regulating their emotions (see Joormann and Quinn, 2014). Furthermore, recent findings suggest that cognitive bias modification strategies to reduce cognitive biases can attenuate biological reactivity to stress (Jopling et al., 2020) and mood lability (Hoorelbeke et al., 2015). However, it is also possible that these results are unique to the current sample of unselected participants, many of whom had subclinical levels of depression (Radloff, 1977). In a clinical sample, we might observe that these same variables predict a more perpetual pattern of brooding, evidenced by less decline in brooding after the exam.

Although this study is the first to assess the relative contribution of deficits in different components of cognitive control to rumination, it is not without its limitations. First, although use of a longitudinal design provides us with the benefit of establishing the chronological order of events, it does not allow a casual inference. Hence, future studies should also utilize experimental designs to strengthen the evidence provided by this study. Second, the current study was designed to examine predictors of rumination in an unselected sample in everyday life. We chose to focus on a university sample because we were able to assess rumination in response to a ubiquitous naturalistic stressor (i.e., midterm exam), but the results of this study might not be generalizable to other populations and other stressors. Therefore, future studies should replicate these results in order to test the generalizability of our findings.

There are several exciting avenues of future research that might be considered. While in this study we aimed to contribute to the existing literature by assessing the association of cognitive control with state rumination, it is important for future research to clarify the role of cognitive control in trait rumination as well as in state rumination in response to different types of stressors. The duration, intensity, or underlying emotions elicited in the stressor (e.g., sadness, anxiety, or anger) might alter the association between cognitive control and rumination. It is possible, for example, that the tendency to ruminate in response to intense acute stressors, such as a midterm exam, has different underlying cognitive processes than the tendency to ruminate in response to smaller day-to-day stressors. Thus, future studies should elucidate the difference between state and trait rumination in their association with cognitive control components and biases. In addition, future studies should assess the role of discarding in relation to rumination. We assessed the three component of cognitive control (inhibition, shifting, and updating) posited by Miyake and Friedman (2012). However, Friedman and Miyake (2004) also suggested that inhibition is an overextended category, and should perhaps be broken down to different components. Consistent with this recommendation, Zetsche et al. (2018) separated discarding from inhibition in their meta-analysis, and found particularly promising evidence of an association between discarding and rumination. Thus, although the current study did not assess discarding in relation to rumination, it will be important for future research to examine the unique effects of discarding over and above other components of cognitive control.

The results of this study have important empirical and theoretical contributions. We found that brooding, and not reflection, predicted negative affect at each future time point. These findings demonstrate the short window between rumination and depressive symptoms, and highlight the need for early clinical interventions that focus on reducing rumination. Furthermore, our findings inform cognitive models of rumination by elucidating the aspects of cognitive control that predict rumination. They also shed light on the cognitive underpinnings of maladaptive (i.e., brooding) versus adaptive (i.e., reflection) forms of rumination. Given that more negative and less positive shifting biases predicted higher levels of brooding immediately after the stressor, negative shifting biases might serve as a target for future experimental research and, if replicated, for clinical interventions. Cognitive interventions designed to reduce deficits in controlling negative information in working memory have attenuated levels of rumination (Siegle et al., 2007), depressive symptoms (Siegle et al., 2007; Wells and Beevers, 2010), and biological responses to stress (Jopling et al., 2020; LeMoult, 2020). Applying the results of this study to that work suggests that targeting negative shifting biases specifically could increase the effectiveness of these interventions.

## Data Availability Statement

The raw data supporting the conclusions of this article will be made available by the authors, without undue reservation.

## Ethics Statement

The studies involving human participants were reviewed and approved by The University of British Columbia Behavioral Research Ethics Board (BREB). The patients/participants provided their written informed consent to participate in this study.

## Author Contributions

BZ contributed to formal analysis, data curation, writing the original draft, and reviewing and editing. JW contributed to methodology, programing cognitive tasks, validation, investigation, reviewing and editing, and project administration. JL contributed to conceptualization, methodology, programing cognitive tasks, validation, resources, reviewing and editing, supervision, and funding acquisition. All authors agreed to be accountable for the content of the work.

## Conflict of Interest

The authors declare that the research was conducted in the absence of any commercial or financial relationships that could be construed as a potential conflict of interest.

## Footnotes

1 http://www.macbrain.org/faces/index.htmt
