# Corrigendum: Cognitive control and ruminative responses to stress: understanding the different facets of cognitive control

**DOI:** 10.3389/fpsyg.2023.1195675

**Published:** 2023-06-09

**Authors:** Bita Zareian, Jessica Wilson, Joelle LeMoult

**Affiliations:** Depression, Anxiety and Stress Lab, Department of Psychology, The University of British Columbia, Vancouver, BC, Canada

**Keywords:** rumination, cognitive control, inhibition, shifting, updating, depression

The authors identified an error in one of the syntax files that was used to calculate switch costs from the Affective Switching Task (Genet et al., 2013). One of the syntax files assigned the value “−99” to the missing and inaccurate trials without eliminating them from further analysis. This affected ~10% of the trials, and the values of non-affective positive switch cost, non-affective negative switch cost, affective positive switch cost, and affective negative switch cost. These changes did not impact the results of Aim 1 analyses and had minor impacts on the results of the Aim 2 analyses. Specifically, while the values of all coefficients for the Aim 2 analyses change, the significance of results remained the same, except for the *p*-values for the non-affective negative switch costs as a predictor of the slopes of brooding and the second slope of reflection. Correction of this error changes the significance of 3 out of 72 predictors across the two HLM models and excludes one participant from Aim 2 analyses due to the inadequate number of accurate trials in the Affective Switching Task. The interpretation of the results and conclusions remains the same.

Corrections have been made to *Main analyses, Brooding, Paragraphs 1* and *2*. The corrected text are shown below.

As expected, higher levels of brooding immediately after the exam were predicted by higher levels of depression at baseline, *B* = 0.932, *t*(174) = 2.93, *p* = 0.004, *R*^2^ = 0.03. Furthermore, higher levels of brooding immediately after the exam were predicted by less positive shifting bias, more negative shifting bias, less positive inhibition bias, and less inhibition of neutral stimuli at baseline. Specifically, higher levels of brooding were associated with *faster* switching away from positive (i.e., less positive shifting bias), *B* = −0.759, *t*(174) = −2.28, *p* = 0.024, *R*^2^ = 0.02, and *slower* switching away from negative (i.e., more negative shifting bias), *B* = 0.675, *t*(174) = 2.57, *p* = 0.011, *R*^2^ = 0.02. In addition, higher levels of brooding immediately after the exam were associated with greater inhibition of positive (i.e., preventing positive words from entering working memory or positive inhibition bias), *B* = −1.828, *t*(174) = −2.34, *p* = 0.021, *R*^2^ = 0.02, and less inhibition of neutral (i.e., preventing neutral words from entering working memory), *B* = 1.536, *t*(174) = 2.18, *p* = 0.031, *R*^2^ = 0.01.

The slope of decline in brooding from immediately after the exam until the second follow-up, which was on average 8 h after the exam, was predicted by similar baseline cognitive variables that predicted the initial level of brooding, but in the opposite direction: more sustained brooding was associated with more positive shifting bias, more positive inhibition bias, and more inhibition of neutral stimuli. Specifically, a flatter slope of decline in brooding was associated with slower switching away from positive (i.e., positive shifting bias), *B* = 0.257, *t*(174) = 3.12, *p* = 0.002, *R*^2^ = 0.1, and more difficulty inhibiting positive (i.e., positive inhibition bias), *B* = 0.409, *t*(174) = 2.12, *p* = 0.036, *R*^2^ = 0.04. A flatter slope of decline in brooding was also associated with more inhibition of neutral, *B* = −0.369, *t*(174) = −2.25, *p* = 0.026, *R*^2^ = 0.04. The slope of change after the second follow-up was not predicted by any of the cognitive variables. Adding the time between the baseline session and the exam as a covariate did not change the results.

Corrections have been made to *Main analyses, Reflection, Paragraph 1*. The corrected text is shown below.

Reflection right after the exam was predicted by only difficulty inhibiting neutral stimuli, *B* = 1.534, *t*(174) = 2.38, *p* = 0.018, *R*^2^ = 0.02, such that greater reflection immediately after the exam was associated with less inhibition of neutral. The slope of change in reflection from immediately after the exam until the second follow-up, which was on average 8 h after the exam, was associated with positive shifting bias, *B* = 0.218, *t*(174) = 2.91, *p* = 0.004, *R*^2^ = 0.10, and inhibition of neutral stimuli, *B* = −0.526, *t*(174) = −3.39, *p* < 0.001, *R*^2^ = 0.11, such that a flatter slope of decline in reflection was associated with slower switching away from positive and less difficulty inhibiting neutral. The slope of change after the second follow-up was predicted by negative shifting bias *B* = −0.010, *t*(174) = −2.28, *p* = 0.024, *R*^2^ = 0.01. Adding the time between the baseline session and the exam as a covariate did not change the results.

Corrections have been made to *Discussion, Paragraph 5*. The corrected paragraph is shown below.

We also found that the trajectory of change in brooding was predicted by the variables that predicted the level of brooding right after the exam, but in the opposite direction: faster recovery of brooding from the time of the exam to the second follow-up was predicted by more difficulty inhibiting neutral information, less difficulty inhibiting positive information, and less positive shifting biases.

Corrections have been made to *Supplementary Material, Page 3, Paragraph 2*.

The correct paragraph appears below:

**“General switch cost**. We calculated each participants' average RT on switch trials (*M* = 1475.17, *SD* = 314.63) and repetition trials (*M* = 1334.71, *SD* = 251.22). A *t*-test assessing the difference between the average RT on switch trials versus repetition trials provided evidence for the expected switch cost, *t*(249) = 21.18, *p* < 0.001. We calculated the switch cost by subtracting the average RT for repetition trials from switch trials.”

Lastly, in the published article, there were errors in Table 2, Table 3, and Table 4. The corrected Table 2, Table 3, and Table 4 and their captions appear below.

**Table 2:** Correlation table for cognitive variables.

	**Cognitive variable**	***M (SD*)**	**Correlation**										
			**1**	**2**	**3**	**4**	**5**	**6**	**7**	**8**	**9**	**10**	**11**
1	Affective positive switch cost	128.00 (175.40)	–										
2	Affective negative switch cost	252.54 (176.71)	0.246^**^	–									
3	Non-affective positive switch cost	133.28 (229.51)	0.014	0.276^**^	–								
4	Non-affective negative switch cost	65.81 (180.25)	0.050	0.152^*^	−0.076	–							
5	Break-happy	1,109.90 (118.01)	0.082	0.083	0.099	0.047	–						
6	Break-neutral	1,106.29 (118.88)	0.064	0.069	0.106	0.070	0.745^**^	–					
7	Break-sad	1,119.62 (116.00)	0.069	−0.009	0.078	0.074	0.710^**^	0.769^**^	–				
8	Stroop-negative	627.29 (87.49)	0.103	0.104	0.019	0.082	0.272^**^	0.199^**^	0.211^**^	–			
9	Stroop-neutral	628.38 (82.45)	0.134^*^	0.128^*^	0.033	0.041	0.252^**^	0.173^**^	0.181^**^	0.918^**^	–		
10	Stroop-positive	630.29 (85.67)	0.087	0.124	0.025	0.094	0.288^**^	0.213^**^	0.232^**^	0.909^**^	0.902^**^	–	
11	Stroop-threat	640.52 (89.81)	0.096	0.125^*^	0.032	0.086	0.266^**^	0.179^**^	0.209^**^	0.915^**^	0.894^**^	0.914^**^	–

* p < 0.05.

** p < 0.01.

**Table 3:** Predicting the level and trajectory of brooding.

	**Coeff**	**SE**	***t*(174)**	***p*-value**
**Intercept**				
Intercept	**10.734**	**0.300**	**36.31**	**<0.001**
Baseline depression	**0.932**	**0.318**	**2.93**	**0.004**
Stroop-negative	−0.836	0.781	−1.07	0.286
Stroop-neutral	**1.536**	**0.705**	**2.18**	**0.031**
Stroop-positive	**−1.828**	**0.783**	**−2.34**	**0.021**
Stroop-threat	1.566	0.971	1.61	0.109
Affective positive switch cost	0.364	0.295	1.23	0.219
Affective negative switch cost	0.133	0.281	0.47	0.637
Non-affective positive switch cost	**−0.759**	**0.333**	**−2.28**	**0.024**
Non-affective negative switch cost	**0.675**	**0.262**	**2.57**	**0.011**
Break-happy	0.045	0.492	0.09	0.927
Break-neutral	−0.110	0.523	−0.21	0.833
Break-sad	0.043	0.451	0.09	0.925
**Slope of change until follow-up 2**				
Intercept	**−0.450**	**0.075**	**−6.00**	**<0.001**
Baseline depression	0.010	0.070	0.14	0.891
Stroop-negative	0.186	0.201	0.92	0.357
Stroop-neutral	**−0.369**	**0.164**	**−2.25**	**0.026**
Stroop-positive	**0.409**	**0.193**	**2.12**	**0.036**
Stroop-threat	−0.312	0.268	−1.16	0.246
Affective positive switch cost	−0.069	0.071	−0.98	0.328
Affective negative switch cost	0.019	0.055	0.34	0.734
Non-affective positive switch cost	**0.257**	**0.083**	**3.12**	**0.002**
Non-affective negative switch cost	−0.092	0.060	−1.53	0.127
Break-happy	−0.067	0.102	−0.65	0.514
Break-neutral	0.186	0.118	1.57	0.117
Break-sad	−0.099	0.101	−0.98	0.331
**Slope of change after follow-up 2**				
Intercept	**−0.026**	**0.005**	**−5.04**	**<0.001**
Baseline depression	0.002	0.008	0.24	0.814
Stroop-negative	0.020	0.016	1.24	0.217
Stroop-neutral	−0.023	0.013	−1.79	0.075
Stroop-positive	0.017	0.016	1.02	0.307
Stroop-threat	−0.014	0.019	−0.70	0.484
Affective positive switch cost	−0.001	0.004	−0.15	0.883
Affective negative switch cost	0.002	0.005	0.42	0.672
Non-affective positive switch cost	−0.003	0.005	−0.52	0.608
Non-affective negative switch cost	−0.009	0.006	−1.50	0.136
Break-happy	0.001	0.010	0.12	0.907
Break-neutral	−0.007	0.008	−0.80	0.424
Break-sad	0.003	0.008	0.42	0.674

The bolded values indicate that the values are statistically significant.

**Table 4:** Predicting the level and trajectory of reflection.

	**Coeff**	**SE**	***t*(174)**	***p-*value**
**Intercept**				
Intercept	**9.307**	**0.266**	**34.93**	**<0.001**
Baseline depression	0.448	0.275	1.63	0.105
Stroop-negative	−0.720	0.679	−1.06	0.291
Stroop-neutral	**1.534**	**0.644**	**2.38**	**0.018**
Stroop-positive	−1.055	0.737	−1.43	0.154
Stroop-threat	0.943	0.904	1.04	0.298
Affective positive switch cost	0.016	0.298	0.05	0.957
Affective negative switch cost	0.319	0.257	1.24	0.217
Non-affective positive switch cost	−0.336	0.290	−1.16	0.248
Non-affective negative switch cost	0.454	0.238	1.91	0.058
Break-happy	0.232	0.386	0.60	0.549
Break-neutral	−0.502	0.436	−1.15	0.251
Break-sad	0.396	0.373	1.06	0.290
**Slope of change until follow-up 2**				
Intercept	**−0.315**	**0.071**	**−4.40**	**<0.001**
Baseline depression	0.039	0.069	0.57	0.568
Stroop-negative	0.303	0.192	1.58	0.116
Stroop-neutral	**−0.526**	**0.155**	**−3.39**	**<0.001**
Stroop-positive	0.334	0.206	1.62	0.106
Stroop-threat	−0.321	0.256	−1.26	0.211
Affective positive switch cost	−0.039	0.073	−0.54	0.591
Affective negative switch cost	−0.020	0.057	−0.36	0.719
Non-affective positive switch cost	**0.218**	**0.075**	**2.91**	**0.004**
Non-affective negative switch cost	−0.029	0.061	−0.47	0.637
Break-happy	0.023	0.096	0.24	0.810
Break-neutral	0.157	0.104	1.50	0.135
Break-sad	−0.155	0.098	−1.59	0.115
**Slope of change after follow-up 2**				
Intercept	**−0.022**	**0.004**	**−5.56**	**<0.001**
Baseline depression	−0.004	0.004	−0.96	0.339
Stroop-negative	0.012	0.012	0.98	0.327
Stroop-neutral	−0.010	0.009	−1.07	0.285
Stroop-positive	0.018	0.014	1.25	0.212
Stroop-threat	−0.015	0.013	−1.14	0.254
Affective positive switch cost	−0.001	0.004	−0.27	0.785
Affective negative switch cost	0.005	0.004	1.32	0.187
Non-affective positive switch cost	−0.004	0.004	−1.09	0.278
Non-affective negative switch cost	**−0.010**	**0.004**	**−2.28**	**0.024**
Break-happy	−0.002	0.008	−0.29	0.773
Break-neutral	−0.002	0.006	−0.37	0.710
Break-sad	0.001	0.006	0.15	0.878

The bolded values indicate that the values are statistically significant.

This correction was initiated by the authors in compliance with the open science practices and to ensure the integrity of future literature reviews and meta-analytic work that might draw conclusions from the results of this manuscript. The authors apologize for these errors and state that they do not change the scientific conclusions of the article in any way. The original article has been updated.
