# Supplementary material for: Cognitive Control and Ruminative Responses to Stress: Understanding the Different Facets of Cognitive Control
Source: Front Psychol. 2021 May 7;12:660062. doi: 10.3389/fpsyg.2021.660062 (PMC8138047; doi:10.3389/fpsyg.2021.660062)
Supplement: Supplementary file 1 [file Table_1.DOCX]

**Supplemental Online Materials**

**Content:**

**Supplemental Methods and Results:**

1. Negative mood indication
2. Additional information about experience sampling method
3. Emotional Stroop Task
4. Affective Switching Task
5. Data cleaning
6. Model selection
7. HLM model for aim 2
8. R code for calculating *R^2^*
9. Additional HLM model: n-back variables as sole predictor of rumination

**Supplemental Methods and Results**

**1 Negative mood induction**

A series of paired sample *t*-test showed that mood inductions were successful in increasing negative affect and reducing positive affect. See the following table for the results.

Table S1. Positive and negative affect pre- and post-movies

|  | Negative affect  *M (SD)* | *t*(*df*) | *p*-value | Positive affect  *M (SD)* | *t*(*df*) | *p*-value |
| --- | --- | --- | --- | --- | --- | --- |
| Pre-Movie 1 | 16.67 (6.34) | -11.73 (264) | < .001 | 11.03 (3.28) | 12.88 (263) | < .001 |
| Post-Movie 1 | 20.76 (7.75) |  |  | 8.63 (3.14) |  |  |
| Pre-Movie 2 | 17.11 (7.13) | -7.27 (264) | < .001 | 8.13 (3.13) | 8.72 (265) | < .001 |
| Post-Movie 2 | 19.67 (7.56) |  |  | 6.98 (2.26) |  |  |
| Pre-Movie 3 | 16.48 (7.33) | -11.51 (257) | < .001 | 7.42 (3.07) | 5.42 (263) | < .001 |
| Post-Movie 3 | 20.66 (8.19) |  |  | 6.49 (2.25) |  |  |
| Pre-Movie 4 | 15.23 (6.83) | 9.710 (254) | < .001 | 8.09 (3.33) | -12.96 (259) | < .001 |
| Post-Movie 4 | 13.31 (5.93) |  |  | 10.00 (3.78) |  |  |

*Movies 1, 2, and 3 were used to induce negative affect and movie 4 was used to bring participants’ negative and positive affect back to the baseline.*

**2 Additional Information about experience sampling method**

Table S2. The average time between follow-up surveys.

|  | *M* | *SD* |
| --- | --- | --- |
| Midterm – Follow up 1 | 1.96 | 1.16 |
| Follow up 1 – Follow up 2 | 5.63 | 4.89 |
| Follow up 2 – Follow up 3 | 7.15 | 5.70 |
| Follow up 3 – Follow up 4 | 6.62 | 5.63 |
| Follow up 4 – Follow up 5 | 3.65 | 2.76 |
| Follow up 5 – Follow up 6 | 6.17 | 5.34 |
| Follow up 6 – Follow up 7 | 6.65 | 5.51 |
| Follow up 7 – Follow up 8 | 7.37 | 5.76 |
| Follow up 8 – Follow up 9 | 10.27 | 16.35 |

**2 Inhibiting: Emotional Stroop Task**

As expected, the average reaction time (RT) for trials with colour words in which the text and colour matched (e.g., “blue” was written in blue font) was significantly faster than those in which the text and colour did not match (*M* = 619.35, *SD* = 118.08 and *M* = 680.69, *SD* = 7.48, respectively), *t*(248) = 12.47, *p* < .0001. The average reaction time (RT) for negative trials was 627.29 (*SD* = 87.48), for neutral trials was 628.38 (*SD* = 82.45), for positive trials was 630.29 (*SD* = 85.67), and for threat trials was 640.52 (*SD* = 89.81).

**3 Shifting: Affective switching task**

**General switch cost.** We calculated each participants’ average RT on switch trials (*M* = 1299.27, *SD* = 344.59) and repetition trials (*M* = 1193.08, *SD* = 284.99). A *t*-test assessing the difference between the average RT on switch trials versus repetition trials provided evidence for the expected switch cost, *t*(266) = 15.17, *p* < .0001. We calculated the switch cost by subtracting the average RT for repetition trials from switch trials.

**Valence-specific switch costs.** Valence-specific switch costs were calculated by taking into account the rule being switched to as well as the valence of the image. For example to calculate the switch cost associated with switching to the affective rule on a positive target image, we subtracted the average RT of repetition trials in which the affective rule is repeated while categorizing a positive target image from the average RT of all switch trials in which the rule switched from non-affective to affective while categorizing a positive target image. Using this procedure, four different specific switch costs were calculated: switch cost to affective rule while categorizing positive picture (affective positive switch), switch cost to non-affective rule while categorizing positive picture (non-affective positive switch), switch cost to affective rule while categorizing negative pictures (affective negative switch), and switch cost to non-affective rule while categorizing negative picture (non-affective negative switch).

**4 Data cleaning**

**Emotional Stroop Task.** Consistent with Gilboa-Schechtman, Revelle, and Gotlib (2000) and Dresler at al. (2009), we eliminated inaccurate trials and trials on which the reaction time (RT) was two standard deviations above or below the mean. We also eliminated data from participants whose accuracy on all trials were below chance.

**Affective Switching Task.** Consistent with Genet et al.’s (2013) approach to data cleaning, we first eliminated inaccurate trials. Next, we substituted RTs higher than 2.5 SD from the mean with the value of *M* + 2.5 SD (RT = 3788 ms) and the RTs below 250 ms with the value 250. We also eliminated data from participants whose accuracy on all trials were below chance (Malooly, Genet & Siemer, 2013).

**Emotional 2-Back Task.** Consistent with Levens and Gotlib (2010), we eliminated inaccurate trials and trials with RT 2.5 SD above and below the mean. Given the difficulty of this task, there are no cut offs used to delete participant data based on their total accuracy on the task.

**5 Model selection and data cleaning**

**Brooding.** Following recommendations by Singer and Willett (2003), the piecewise model was developed based on our visual inspection of the data showing that the level of brooding reduces with a steeper slope between the time of the exam and follow-up 2 (i.e., 8 hours after the exam; Change 1), compared to between follow-up 2 and follow-up 9 (Change 2). In comparison to the linear model, both the quadratic, *X^2^*(4) = 196.99, *p* < .001, and the piecewise models, *X^2^*(4) = 1738.36, *p* < .001, were significantly better fits for the data. Given the lower AIC of the piecewise model (AIC = 5550.04) in comparison to the quadratic model (AIC = 7091.41), the piecewise model was used.

**Reflection.** Following recommendations by Singer and Willett (2003), the piecewise model was developed based on our visual inspection of the data showing that the level of reflection reduces with a steeper slope between the time of the exam and follow-up 2 (i.e., 8 hours after the exam; Change 1), compared to between follow-up 2 and follow-up 9 (Change 2). In comparison to the linear model, both the quadratic, *X^2^* (4) = 102.23, *p* < .001, and the piecewise models, *X^2^* (4) = 1718.33, *p* < .001, were significantly better fits for the data. Given the lower AIC of the piecewise model (AIC = 5138.95) in comparison to the quadratic model (AIC = 6755.04), the piecewise model was used.

**6 HLM model for aim 2: Cognitive control biases predict the level and trajectory of brooding and reflection**

We entered the cognitive bias scores as simultaneous predictors at Level 2. For the Affective Stroop Task (i.e., inhibition), we entered the mean RTs for each of the four critical trials (i.e., Stroop-positive, Stroop-negative, Stroop-neutral and Stroop-threat). For the Affective Switching Task (i.e., shifting), we entered the four critical switch-cost variables (i.e., affective positive switch cost, non-affective positive switch cost, affective negative switch cost, non-affective negative switch cost). Finally, for the Emotional 2-Back Task (i.e., updating), we entered the mean RTs for each of the three valenced break-set trials (i.e., break-sad, break-happy, and break-neutral). In addition, to control for symptoms of depression, we added baseline CES-D scores to the Level 2 model.

**Level 1:**

Brooding_ij_ = *β*_0j_ + *β*_1j_ (Change 1) + *β*_2j_ (Change 2) + *e*_tj_

**Level 2:**

*β*_0j_ = *γ*_00_ + *γ*_01_(Stroop-positive_j_) + *γ*_02_(Stroop-negative_j_) + *γ*_03_(Stroop-neutral_j_) + *γ*_04_(Stroop-threat_j_)+ *γ*_05_(affective positive switch cost _j_) + *γ*_06_(non-affective positive switch cost _j_) + *γ*_07_(affective negative switch cost _j_) + *γ*_08_(non-affective negative switch cost _j_) +*γ*_09_( break-sad _j_) + *γ*_010_(break-happy _j_) + *γ*_011_(break-neutral _j_) + *γ*_012_(CES-D _j_)+ *u*_0j_

*β*_1j_ = *γ*_10_ + *γ*_11_(Stroop-positive _j_) + *γ*_12_(Stroop-negative _j_) + *γ*_13_(Stroop-neutral _j_) + *γ*_14_(Stroop-threat _j_)+ *γ*_15_(affective positive switch cost _j_) + *γ*_16_(non-affective positive switch cost _j_) + *γ*_17_(affective negative switch cost _j_) + *γ*_18_(non-affective negative switch cost _j_) +*γ*_19_( break-sad _j_) + *γ*_110_(break-happy _j_) + *γ*_111_(break-neutral _j_) + *γ*_112_(CES-D _j_)+ *u*_1j_

*β*_2j_ = *γ*_20_ + *γ*_21_(Stroop-positive_j_) + *γ*_22_(Stroop-negative_j_) + *γ*_23_(Stroop-neutral_j_) + *γ*_24_(Stroop-threat_j_)+ *γ*_25_(affective positive switch cost _j_) + *γ*_26_(non-affective positive switch cost _j_) + *γ*_27_(affective negative switch cost _j_) + *γ*_28_(non-affective negative switch cost _j_) +*γ*_29_( break-sad _j_) + *γ*_210_(break-happy _j_) + *γ*_211_(break-neutral _j_) + *γ*_212_(CES-D _j_)+ *u*_2j_

**7 R code for calculating *R^2^***

install.packages(“r2glmm”)

library(r2glmm)

r2beta(Model, method = 'sgv') #Model is the name we gave to our HLM model in R

**8 Additional HLM model: n-back variables as sole predictor of rumination**

Table S3. HLM model: break-set trials as predictors of rumination

| Fixed Effect | Coeff | SE | *t* (185) | *p* |
| --- | --- | --- | --- | --- |
| Intercept |  |  |  |  |
| **Intercept** | **19.167** | **0.471** | **40.66** | **<0.001** |
| Break-happy | 0.006 | 0.006 | 1.03 | 0.304 |
| Break-neutral | -0.005 | 0.007 | -0.71 | 0.478 |
| Break-sad | 0.005 | 0.006 | 0.78 | 0.435 |
| Slope of change until follow-up 2 | | | | |
| **Intercept** | **-0.468** | **0.095** | **-4.92** | **<0.001** |
| Break-happy | -0.001 | 0.001 | -0.93 | 0.353 |
| **Break-neutral** | **0.003** | **0.001** | **2.02** | **0.045** |
| Break-sad | -0.002 | 0.001 | -1.70 | 0.091 |
| Slope of change after follow-up 2 | | | | |
| Intercept | -0.052 | 0.008 | -6.56 | <0.001 |
| Break-happy | 0.00002 | 0.0001 | 0.14 | 0.890 |
| Break-neutral | -0.00006 | 0.0001 | -0.61 | 0.544 |
| Break-sad | -0.000005 | 0.0001 | -0.05 | 0.959 |
